# Dual-source dual-power electrospinning and characteristics of multifunctional scaffolds for bone tissue engineering

**DOI:** 10.1007/s10856-012-4669-4

**Published:** 2012-05-17

**Authors:** Chong Wang, Min Wang

**Affiliations:** Department of Mechanical Engineering, The University of Hong Kong, Pokfulam Road, Pokfulam, Hong Kong

## Abstract

Electrospun tissue engineering scaffolds are attractive due to their distinctive advantages over other types of scaffolds. As both osteoinductivity and osteoconductivity play crucial roles in bone tissue engineering, scaffolds possessing both properties are desirable. In this investigation, novel bicomponent scaffolds were constructed via dual-source dual-power electrospinning (DSDPES). One scaffold component was emulsion electrospun poly(d,l-lactic acid) (PDLLA) nanofibers containing recombinant human bone morphogenetic protein (rhBMP-2), and the other scaffold component was electrospun calcium phosphate (Ca–P) particle/poly(lactic-*co*-glycolic acid) (PLGA) nanocomposite fibers. The mass ratio of rhBMP-2/PDLLA fibers to Ca–P/PLGA fibers in bicomponent scaffolds could be controlled in the DSDPES process by adjusting the number of syringes used to supply solutions for electrospinning. Through process optimization, both types of fibers could be evenly distributed in bicomponent scaffolds. The structure and properties of each type of fibers in the scaffolds were studied. The morphological and structural properties and wettability of scaffolds were assessed. The effects of emulsion composition for rhBMP-2/PDLLA fibers and mass ratio of fibrous components in bicomponent scaffolds on in vitro release of rhBMP-2 from scaffolds were investigated. In vitro degradation of scaffolds was also studied by monitoring their morphological changes, weight losses and decreases in average molecular weight of fiber matrix polymers.

## Introduction

With an aging population in our society and with more young people around us who are more physically active than previous generations, there is an increasing demand for bone tissue repair or regeneration due to many more incidents of bone disease or trauma now and in the future. “Bone tissue engineering”, which combines the use of biomaterials with cells and other bone-regeneration stimuli, offers a promising way for the treatment of bone defects [1]. For bone tissue repair using the tissue engineering approach, a three-dimensional (3D) materials matrix is required to provide an appropriate microenvironment for cells to populate on and function well during bone tissue formation [2, 3]. In scaffold-based bone tissue engineering, the combination of a scaffold with living cells and/or biological cues such as growth factors has been shown to be effective to promote bone regeneration [4]. The surface topography and physico-chemical characteristics of scaffolds also contribute to cell adhesion and proliferation and high porosity and highly interconnected porous structure are highly desirable [5, 6]. Since the emergence of tissue engineering more than two decades ago, many materials (mainly polymers) and scaffold fabrication techniques including electrospinning have been investigated [7, 8, 11]. Over the past 10 years, due to structure similarity to the extracellular matrix (ECM) of body tissues, nanofibrous scaffolds electrospun from a variety of biodegradable polymers have been investigated for tissue engineering. These scaffolds exhibit excellent performance in promoting cells to adhere to and spread over the scaffolds as well as further triggering the cells to secrete appropriate proteins targeted to specific tissues or organs. Furthermore, using an appropriate electrospinning technique, bioactive agents such as growth factors can be encapsulated in nanofibrous scaffolds [9, 10], with the resultant scaffolds mimicking the role of ECM on the storage and release of bio-signals secreted by cells which triggers further cell proliferation and differentiation.

For bone tissue engineering, biodegradable polymers [12–14], bioceramics [15, 16] and composites [17, 18] have been fabricated into scaffolds. From the material point of view, bone ECM consists of an organic matrix (mainly collagen) and inorganic, nanosized bone apatite crystals which are arranged in a hierarchical structure [17]. Due to chemical similarity to bone apatite, synthetic, osteocondcutive calcium phosphates are widely used to repair bone defects and bone can be formed in direct contact with these ceramics [19]. The incorporation of calcium phosphate (Ca–P) bioceramics in materials such as polymers to form composites is shown to assist bone mineralization [20, 21]. For bone tissue engineering, it is therefore natural to form electrospun composite scaffolds comprising of a degradable biopolymer and bioactive (osteoconductive) Ca–P nanoparticles [22, 23]. The Ca–P nanoparticles incorporated will render the scaffolds osteoconductive, facilitating osteoblastic cell proliferation and differentiation as well as calcification of bone matrix [24].

Because biological cues play important roles in bone tissue regeneration, controlled delivery of growth factors or other biomolecules in the scaffolds are considered key factors in bone tissue engineering [25, 26]. Growth factors secreted by cells significantly influence different cellular events such as cell adhesion, proliferation and differentiation. Among various growth factors, bone morphogenetic proteins 2 (BMP-2) is generally regarded as the most potent growth factor for bone formation, especially in the osteogenic differentiation of mesenchymal stem cells to osteoblasts [27, 28]. Due to the high surface area-to-volume ratio, electrospun nanofibers are considered excellent vehicles for the storage and controlled release of growth factors. As electrospinning of simple blends of BMP-2 and polymer solutions results in low encapsulation efficiency and high burst release of BMP-2 and risks BMP-2 denaturation, emulsion electrospinning, which can produce core–shell structured fibers, appears to be suitable for constructing tissue engineering scaffolds containing biomolecules [28].

For bone tissue engineering, our previous investigations have shown that: (1) electrospun osteoconductive nanocomposite fibers containing carbonated hydroxyapatite nanoparticles promoted the proliferation and differentiation of osteoblastic cells [23]; (2) the controlled release of recombinant BMP-2 (rhBMP-2) from scaffolds rendered the scaffolds with desired osteoinductivity [29]. Therefore, incorporating both Ca–P nanoparticles and rhBMP-2 molecules in electrospun scaffolds should have a synergetic effect on cell proliferation and differentiation, accelerating bone regeneration. By varying the amounts of Ca–P and rhBMP-2, nanofibrous scaffolds with modulated osteoconductivity and osteoinductivity could be developed. In this investigation, bicomponent scaffolds, which comprised a rhBMP-2 encapsulated poly(d,l-lactic acid) (PDLLA) fibrous component and an Ca–P nanoparticle incorporated poly(lactic-*co*-glycolic acid) (PLGA) fibrous component, were constructed through the use of our dual-source dual-power electrospinning (DSDPES) technique [30, 31]. Amorphous Ca–P, which is degradable and more bioactive than HA, was used as the bioceramic. Ca–P/PLGA composite fibers were electrospun using an established procedure [23] and emulsion electrospinning was employed to incorporate rhBMP-2 in PDLLA [28]. The mass ratio of the two fibrous components was varied in order to obtain bicomponent scaffolds with tunable osteoconductivity and osteoinductivity. Characteristics of electrospun mono- and bicomponent scaffolds were subsequently studied. The in vitro rhBMP-2 release behaviour and in vitro degradation of scaffolds were also investigated.

## Materials and methods

### Materials

Amorphous Ca–P nanoparticles were produced in-house using a previously established process [32]. All chemicals used for synthesizing Ca–P nanoparticles were supplied by reputable manufacturers and the deionized water (DI water) for all experiments was obtained using a DI water producer (Model D12681, Barnstead International, USA). Poly(lactic-*co*-glycolic acid) (PLGA) with a LA:GA molar ratio of 50:50 and poly(d,l-lactic acid) (PDLLA) with an average molecular weight of 120 kDa were purchased from Lakeshore Biomaterials, USA. Sodium dodecyl sulfate (SDS), Span-80 surfactant, phosphate buffered saline (PBS) tablets and bovine serum albumin (BSA) protein were Sigma–Aldrich products. Chloroform and *N*,*N*-dimethylformamide (DMF) were supplied by Uni Chem Co. Dimethyl sulfoxide (DMSO) was supplied by Fisher Scientific Inc, USA. rhBMP-2 with a molecular weight of 26KDa was purchased from Shanghai Rebone Biomaterials, China. The human BMP-2 ELISA kits were purchased from Peprotech Inc., USA. An electrical balance with an accuracy of 0.1 mg was used for weighing materials and samples.

### Methods

#### Scaffold fabrication

##### Emulsion formulation

To construct monocomponent rhBMP-2/PDLLA scaffolds (and also to produce the rhBMP-2/PDLLA fibrous component in bicomponent scaffolds), emulsion electrospinning was used. To form water-in-oil emulsions for emulsion electrospinning, 10 μg of rhBMP-2 and 2 mg of BSA (as a stabilizer) were dissolved in 1 ml of DI water to produce the rhBMP-2 loaded water phase. The oil phase was formed by dissolving 1.5 g of PDLLA and 75 μl of Span-80 (the surfactant for making emulsions, 5 wt% in respect to PDLLA) in 10 ml of chloroform, giving a 15 % w/v polymer solution. The uniform emulsions were produced by dripping the water phase into the oil phase, which was followed by an ultra-sonication treatment in an ice-water bath.

##### Formation of Ca–P/PLGA composite suspension for electrospinning

Nanosized Ca–P was made by rapidly mixing an acetone solution of Ca (NO_3_)_2_·4H_2_O with an aqueous solution of (NH_4_)_2_HPO_4_ at a molar ratio of Ca:P = 1.5:1 using a magnetic stirrer, followed by stirring, centrifugation and washing. The slurry of nanoprecipitates was freeze-dried using a Labconco Free-Zone freezedrying system to obtain dry powder [32]. The Ca–P nanoparticles synthesized were characterized using various techniques. X-ray diffraction (XRD) analysis was performed on an X-ray diffractometer (Raguku Model D/max III, Japan) using Cu Kα radiation and Ni filter. XRD patterns were collected at room temperature over the 2*θ* range of 20–50° at a scanning rate of 2°/min. The morphology, size and structure of Ca–P nanoparticles were studied using a field emission scanning electron microscope (FE-SEM, LEO 1530, Germany) and a transmission electron microscope (FEI Tecnai G2 20 S-TWIN Scanning TEM, the Netherlands). The Ca:P ratio of Ca–P particles was determined using an energy dispersive X-ray spectrometer (EDX) attached to the TEM.

When Ca–P/PLGA suspensions were prepared for composite fiber electrospinning, a two-step method was used to reduce Ca–P particle agglomeration in the suspensions. Briefly, 0.25 g and 1 g of PLGA were dissolved separately in 5 ml of a mixed solvent system (chloroform:DMF = 4:1), forming polymer solution A and solution B, respectively. 138 mg of Ca–P nanoparticles were subsequently added into solution A, which was followed by a 5 min ultra-sonication treatment, producing a good dispersion of Ca–P nanoparticles in the PLGA solution. The Ca–P/PLGA suspension formed was then added to solution B, which was followed by another 5 min ultra-sonication treatment, yielding a uniform Ca–P/PLGA suspension (at 12.5 % w/v polymer concentration) with a composition of 10 wt% Ca–P nanoparticles (in respect to the weight of PLGA).

##### Fabrication of mono- and bicomponent scaffolds through dual-source dual power (DSDP) electrospinning

**Fig. 1:**
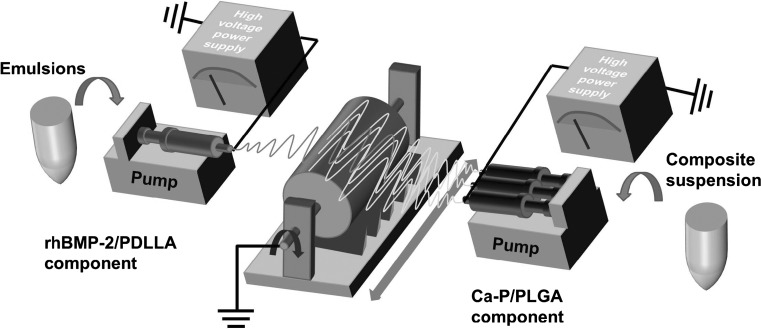
Schematic diagram of the experimental setup for dual-source dual-power electrospinning (DSDPES)

In our previous research, to construct multicomponent fibrous systems for biomedical applications, a multiple-source electrpspinning approach was proposed and in practice, a dual-source dual-power electrospinning (DSDPES) technique was developed for various bicomponent fibrous structures [30, 31]. In the current investigation, DSDPES was employed to construct bicomponent scaffolds containing rhBMP-2/PDLLA and Ca–P/PLGA fibers and the experimental setup is schematically shown in Fig. 1. The DSDPES apparatus comprised two high voltage power supplies, two syringe pumps and a low rotation speed drum as the fiber collector. When the two syringes, which were loaded with an emulsion and a nanocomposite suspension, respectively, were applied separately with high voltages by the two high voltage power supplies, rhBMP-2/PDLLA fibers and Ca–P/PLGA nanocomposite fibers were electrospun simultaneously and collected by the rotating fiber collector to form a non-woven bicomponent scaffold. Furthermore, by varying the number of syringes used for each electrospinning component and hence changing the ratio of syringes between the two electrospinning sources (e.g., from 1:1 to 1:3), the mass ratio of rhBMP-2/PDLLA to Ca–P/PLGA fibers in bicomponent scaffolds would be altered, resulting possibly in different osteoconductivity-to-osteoinductivity ratios of bicomponent scaffolds. The stainless steel needles attached to syringes had an inner diameter of 0.4 mm. The applied voltage, solution feeding rate and working distance for producing rhBMP-2/PDLLA fibers were 10 kV, 1.98 ml/h and 10 cm, respectively, while those for electrpsoinning Ca–P/PLGA composite fibers were 15 kV, 1.98 ml/h and 15 cm, respectively. Table 1 summarizes the composition of bicomponent scaffolds and monocomponent scaffolds (which acted as controls) that were produced in this investigation.

**Table 1:** Composition of solutions and emulsions for mono- and bicomponent scaffolds

Scaffold designation	Scaffold composition			
	rhBMP-2/PDLLA Component		Component ratio (in mass)	Ca–P/PLGA component
	Water phase	Oil phase		
F1	10 μg rhBMP-2 + 2 mg BSA/ml 1.0 ml	10 ml 15 % w/v PDLLA polymer solution 75 μl Span-80	100:0	138 mg Ca–P nanoparticles in 10 ml 12.5 % w/v PLGA solution
F2			0:100	
F3			50:50	
F4			33:66	
F5			25:75	
F6	0.5 ml		50:50	
F7	2.0 ml		50:50	

#### Characterization of fibers and scaffolds

The surface morphology of electrospun fibers and the structure of mono- and bicomponent scaffolds were examined using SEM. All electrospun samples for SEM examinations were placed in desiccators for 48 h to remove solvents and sputter-coated with a thin layer of gold. The average diameter of fibers in each scaffold (or in each fibrous component of bicomponent scaffolds) was determined by selecting randomly 100 fibers in each SEM micrograph and measuring their diameters with the help of a UTHSCSA image tool (which could be downloaded freely from http://compdent.uthscsa.edu/dig/download.html). A transmission electron microscope (Philips EM208s TEM, the Netherlands) was used to study the core–shell structure of emulsion electrospun fibers. Samples for TEM observations were obtained by collecting as-spun fibers on copper grids directly. The diameter ratio between the core and the whole fiber was determined by measuring the diameter of water phase core and that of the whole fiber, also with the help of the UTHSCSA image tool. Using a Nikon Eclipse TE2000-U inverted microscope, fluorescence microscopy was conducted to investigate the distribution of rhBMP-2 in emulsion electrospun fibers, with rhBMP-2 being labeled with rhodamine B. Fourier transform infrared (FTIR) spectroscopic analysis of electrospun fibrous scaffolds was conducted using an FTIR equipment (Spectrum BX FTIR spectrometer from Perkin–Elmer, USA) over a range of 500–4,000 cm^−1^ at a resolution of 2 cm^−1^. To assess the wettability, water contact angles of fibrous scaffolds were measured on a contact angle measuring machine (SL200B, Shanghai Solon Tech Inc Ltd, China) using the sessile drop method with DI water as the liquid. During measurement, square samples (10 mm × 10 mm) were cut from electrospun fibrous membranes and placed on the stage of the measuring machine. The contact angle of the water drop on the sample surface was determined at room temperature following a standardized procedure and using a proprietary software. Three measurements were carried out at different locations of the same sample and the average value was obtained. (For comparison in the wettability study, PLGA and PDLLA thin films were also made using the solvent casting method. Their water contact angles were measured using the same procedure.)

For bicomponent scaffolds produced, the mass ratio between rhBMP-2/PDLLA fibers and Ca–P/PLGA fibers needed to be determined or verified. Due to the structure similarity between PDLLA and PLGA, there is no common solvent that could be used to separate the two types of fibers from each other by dissolving one fibrous component while leaving the other fibrous component intact. Therefore, two methods were used to determine the mass ratio between the two fibrous components. With method one (the “substitution method”), gelatin, a water soluble natural polymer, was used in DSDPES, forming gelatin nanofibers as a substitute for the rhBMP-2/PDLLA fibrous component in bicomponent scaffolds, i.e., for the “substitution method”, bicomponent scaffolds consisting of gelatin fibers and Ca–P/PLGA fibers were constructed via DSDPES. (In this investigation, for electrospinning of gelatin fibers, gelatin was dissolved in acetic acid to form gelatin solutions.) After DSDPES, bicomponent scaffolds formed were placed in desiccators to remove residual solvents. They were then immersed in DI water to remove the gelatin fibers. The bicomponent scaffolds before and after gelatin removal were weighed and hence the mass ratio between rhBMP-2/PDLLA fibers and Ca–P/PLGA fibers could be deduced. With method two (the “TGA method”), the Ca–P contents in mono- and bicomponent scaffolds determined by thermogravimetric analysis (TGA) were used to calculate the mass ratio between the two fibrous components. In the TGA analysis, a thermogravimetric analyzer (Pyris 1 TGA, Perkin–Elmer, USA) was employed and tests were conducted in flowing nitrogen (20 ml/min) environment. (The accuracy of the balance in the TGA equipment was 0.0001 mg). Form the TGA curves obtained, the Ca–P content of monocomponent Ca–P/PLGA scaffolds was determined to be *C*_*0*_ while the Ca–P content of bicomponent scaffolds was *C*. The mass ratio between the two fibrous components (*W*_1_:*W*_2_) for bicomponent scaffolds was calculated using the equation below (*W*_1_ being the weight of rhBMP-2/PDLLA fibers and *W*_2_ being the weight of Ca–P/PLGA fibers):

1 $$ \frac{W_{1}}{W_{2}} = \frac{{1 - {\raise0.7ex\hbox{$C$} \!\mathord{\left/ {\vphantom {C {C_{0}}}}\right.\kern-\nulldelimiterspace} \!\lower0.7ex\hbox{${C_{0}}$}}}}{{{\raise0.7ex\hbox{$C$} \!\mathord{\left/ {\vphantom {C {C_{0}}}}\right.\kern-\nulldelimiterspace} \!\lower0.7ex\hbox{${C_{0}}$}}}} $$

#### Encapsulation efficiency and in vitro release of rhBMP-2

The encapsulation efficiency (EE, %) of rhBMP-2 in rhBMP-2/PDLLA fibers for mono- and bicomponent scaffolds was calculated using the equation below:

2 $$ EE = \frac{A}{{A_{0} }} \times 100\% $$

where *A* is the actual amount of rhBMP-2 encapsulated in each scaffold and *A*_*0*_ is the amount of rhBMP-2 used for encapsulation in each scaffold. The actual amount of rhBMP-2 encapsulated was determined using the extraction method. Briefly, a scaffold sample (at around 20 mg) was dissolved in 2 ml DMSO. 20 ml 0.05 M NaOH with 0.5 % SDS were then added to form a mutually soluble mixture which was transparent. Subsequently, 100 μl of the emulsion were pipetted and the concentration of rhBMP-2 was measured using human BMP-2 ELISA Kit Assay. And the total amount of encapsulated rhBMP-2 in each scaffold sample was determined and the rhBMP-2 encapsulation efficiency was calculated for the scaffold.

The in vitro rhBMP-2 release profiles for mono- and bicomponent scaffolds were determined as follows. Preweighed samples cut from electrospun scaffolds (scaffolds F1 to F5) were immersed in individual centrifuge tubes containing 2 ml of PBS (at pH7.4). 0.02 % sodium azide (bacteriostatic agent), 0.05 % Tween-20 (to reduce the non-specific protein absorption), 0.5 % BSA and 0.1 % heparin were added to the PBS. The tubes with samples were kept in a thermostated shaking water bath which was maintained at 37 °C. At preset times, 0.4 ml of the immersion medium was removed from each tube and frozen at −20 °C for further analysis, and 0.4 ml of fresh PBS was added to each tube for continuing the incubation. The frozen immersion medium was thawed and its rhBMP-2 content was measured using the human BMP-2 ELISA Kit Assay. The amount of rhBMP-2 released at the pre-set time was thus determined and then used for plotting release curves.

#### In vitro fiber degradation

In vitro degradation of mono- and bicomponent scaffolds was investigated for up to 8 weeks in this study. Triplicate samples with an initial individual weight of *M*_*i*_ (at around 20 mg per piece) were cut from electrospun scaffolds (F1 to F5 scaffolds) The samples were then put into 15 ml centrifuge tubes individually and immersed in 3 ml of PBS (pH7.4). The sealed centrifuge tubes were placed in a thermostated shaking water bath which was maintained at 37 °C. The immersion medium was changed every 3 days. At preset times during in vitro degradation tests, immersed samples were taken out of the centrifuge tubes, rinsed in DI water and dried in desiccators. The weight of samples (*M*_*d*_) was then measured using an analytical balance. The mass loss percentage of samples, *M* (%), was calculated using the following equation:

3 $$ M(\% ) = \frac{{M_{i}} - {M_{d}}}{{M_{i}}} \times 100\% $$

The average molecular weight of the matrix polymer of fibers was determined using the light scattering method and a Nano-ZS instrument (Malvern Instruments, UK). The fiber morphology and scaffold structure of samples after PBS immersion for different periods of times were examined using SEM.

#### Statistics analysis

For tests giving out numerical values, the test results were expressed as mean ± standard deviation (SD). For results obtained from different groups or different time points, statistical analysis was performed using ANOVA with a Student’s *t*-test. A value of *p* < 0.05 was considered to be statistically significant.

## Results

### Monocomponent scaffolds

**Fig. 2:**
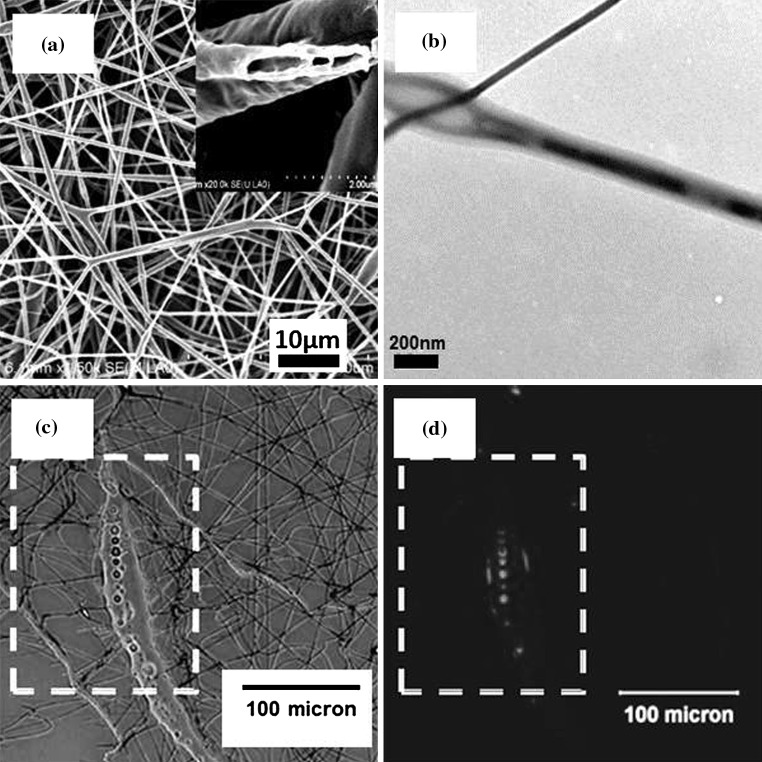
Emulsion electrospun rhBMP-2/PDLLA scaffolds: **a** SEM micrograph showing fiber and scaffold morphology, **b** TEM micrograph of fiber core–shell structure, **c** optical micrograph rhBMP-2/PDLLA scaffold containing RhB-labeled rhBMP-2, and **d** fluorescence micrograph showing the distribution of rhBMP-2 in fibers

Monocomponent rhBMP-2/PDLLA scaffolds were produced via emulsion electrospinnig using an established procedure [28]. The morphology and structure of rhBMP-2/PDLLA fibers and the scaffolds (F1 scaffolds) are shown in Fig. 2. It can be seen from Fig. 2a that fibrous scaffolds with a smooth fiber surface could be electrospun from water-in-oil emulsions formulated. The average diameter of F1 scaffolds was determined to be 570 ± 130 nm. The hollow structure observed on the cross-section of F1 fibers (inset in Fig. 2a) indicated that during emulsion electrospinning, the core–shell structure could be formed owing to the stretch, in the axial direction, and coalescence, mostly in the transverse direction, of water droplets within the emulsion jet. The core–shell structure was also revealed under the TEM examination of fibers and TEM images of fibers displayed a continuous dark core (the water phase) and a light-grey polymeric shell (Fig. 2b). The continuous water phase core would serve as a reservoir for rhBMP-2 molecules and this type of core–shell structured fibers was expected to be good delivery vehicles for biomolecules and provide a controlled release of the biomolecules. To determine the distribution of rhBMP-2 within F1 fibers, RhB-labeled rhBMP-2 was used in formulating emulsions for electrospinning, and the fluorescence signals emitted by RhB-labeled rhBMP-2 in resultant F1 fibers were detected under fluorescence microscopy. The labeling of rhBMP-2 by RhB followed Lochmann et al.’ protocol [33] and the emulsions prepared had the same compositions as those with non-labeled rhBMP-2. Because the diameters of fibers in F1 scaffolds obtained through normal emulsion electrospinning were far too small for investigating rhBMP-2 distribution under the fluorescence microscope, much thicker fibers with diameters around 30 μm were electrospun and collected on cover slips for fluorescence microscopy. In these thicker fibers, the water phase was not continuous (Fig. 2c) owing to insufficient stretching of the polymer jet during electrospinning. But with these thicker fibers, whether the RhB-labeled rhBMP-2 was in the water phase or in the polymer shell or in both could be revealed. It can be seen from Fig. 2c (optical image) and d (fluorescence image) that most of the fluorescence signals (red in color) originated from the water phase (the water droplets) in the fiber, suggesting that the water soluble growth factor rhBMP-2 was contained in the water phase and well protected by the DI water from the polymer solution during the electrospinning process.

**Fig. 3:**
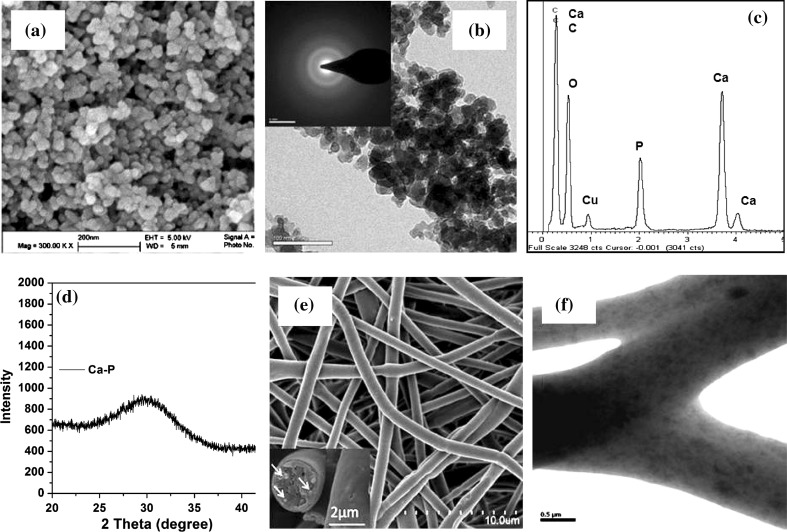
Ca–P nanoparticles and electrospun Ca–P/PLGA nanocomposite scaffolds: **a** SEM micrograph of Ca–P nanoparticles, **b** TEM micrograph and SAD pattern of Ca–P nanoparticles, **c** EDX spectrum of Ca–P nanoparticles, **d** XRD pattern of Ca–P nanoparticles, **e** SEM micrograph showing fiber and scaffold morphology, and **f** TEM micrograph of nanocomposite fiber structure

To fabricate Ca–P/PLGA nanocomposite scaffolds, Ca–P nanoparticles were made and their morphology and structure were studied. It can be seen from Fig. 3a and b that the Ca–P nanoparticles produced in-house were spherical in shape and had diameters around 30 nm. Both selected area diffraction (SAD) patterns obtained through TEM (inset in Fig. 3b) and XRD patterns (Fig. 3d) indicated that the Ca–P nanoparticles were in the amorphous state. EDX spectra obtained during TEM analysis of Ca–P nanoparticles revealed the presence of Ca, P, O, Cu and C (Fig. 3c). The Cu and C peaks arose from the Cu grid which was used for holding Ca–P nanoparticulate samples for TEM examinations and the Cu grid was covered with a C film. O was contained in Ca–P particles. Ca and P were dominant peaks on EDX spectra. The atomic ratio between Ca and P determined in EDX was 1.48 (Fig. 3c), which was close to that of tricalcium phosphate (TCP, with a Ca:P ratio of 1.5). The morphology and structure Ca–P/PLGA nanocomposite fibers and scaffolds (F2 scaffolds) were studied. It can be seen from Fig. 3e that with a fiber nominal composition of 10 wt% Ca–P nanoparticles, Ca–P/PLGA nanocomposite scaffolds with a fiber diameter of 1,140 ± 170 nm could be successfully fabricated. The Ca–P nanoparticles were mainly incorporated in the fibers (inset in Fig. 3e) and a small number of Ca–P nanoparticles were embedded near the fiber surface, resulting in a relatively rough fiber surface. TEM analysis indicated that the incorporated Ca–P nanoparticles had a good dispersion in the fibers (Fig. 3f, the dark particles in the polymer matrix of fibers). TGA analysis of F2 scaffolds showed that the actual Ca–P content of Ca–P/PLGA nanocomposite fibers was 9.5 ± 0.3 %, which was very close to the nominal Ca–P content.

### Bicomponent scaffolds

**Fig. 4:**
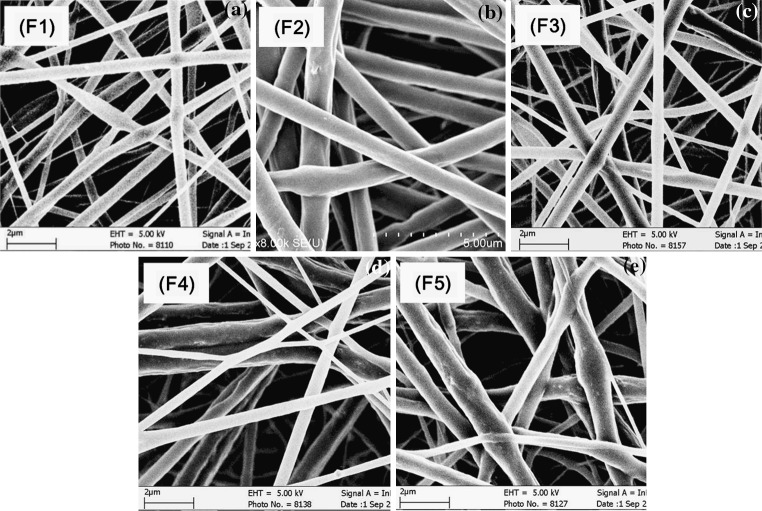
Morphology of fibers and porous structure of mono- and bicomponent scaffolds: **a** F1 monocomponent scaffold (rhBMP-2/PDLLA), **b** F2 monocomponent scaffold (Ca-P/PLGA), **c** F3 bicomponent scaffold, **d** F4 bicomponent scaffold, **e** F5 bicomponent scaffold

DSDPES for fabricating bicomponent scaffolds is schematically illustrated by Fig. 1 and the morphology of electrospun scaffolds (F1 to F5 scaffolds, which were produced using the same electrospinning parameters but differed in the fiber component ratios) is shown in Fig. 4. The SEM micrographs (Fig. 4a–e) indicated that with our DSDPES experimental setup and procedure, the two types of fibers could be evenly distributed in bicomponent scaffolds and that when the mass ratio between the two fibrous components was varied by changing the number of the syringes on each electrospinning source, the uniform distributions of the two types of fibers in bicomponent scaffolds was not altered. F1 to F5 scaffolds had the average fiber diameter of 560 ± 130 nm, 1,170 ± 170 nm, 640 ± 200 nm, 750 ± 280 nm and 850 ± 340 nm, respectively, suggesting that with an increase in mass ratio between Ca–P/PLGA fibers and rhBMP-2/PDLLA fibers, the average fiber diameter of bicomponent scaffolds increased gradually. By changing the number of the syringes on each electrospinning source in DSDPES, the composition, i.e., the mass ratio between the two fibrous components, of bicomponent scaffolds was changed. Both the “substitution method” and the “TGA method” for determining the actual mass ratio of fibrous components gave similar results (Table 2), which were not far from the intended compositions for bicomponent scaffolds.

**Table 2:** Mass ratio of the two fibrous components in bicomponent scaffolds

Scaffold designation	Ratio for number of syringes for rhBMP-2/PDLLA and Ca–P/PLGA	Measured mass ratio between rhBMP-2/PDLLA and Ca–P/PLGA components	
		Substitution method	TGA method
F3	1:1	1:(0.97 ± 0.02)	1:(0.95 ± 0.02)
F4	1:2	1:(1.74 ± 0.06)	1:(1.80 ± 0.03)
F5	1:3	1:(2.54 ± 0.08)	1:(2.70 ± 0.06)

**Fig. 5:**
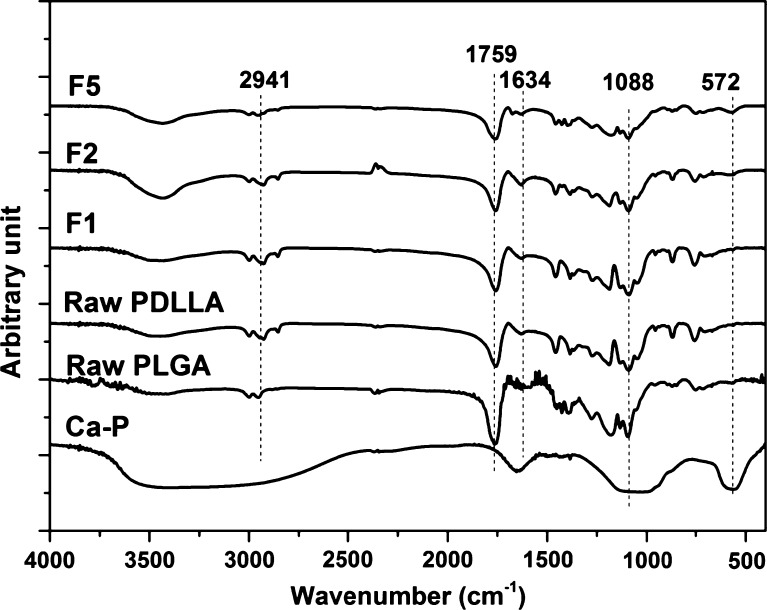
FTIR spectra of raw materials for scaffolds and electrospun scaffolds

FTIR spectra for mono- and bicomponent scaffolds as well as those for Ca–P nanoparticles and PLGA raw material are shown in Fig. 5. Absorption bands for the phosphate group at approximately 572 and 1,435 cm^−1^ were present in the FTIR spectra for Ca–P nanoparticles, Ca–P/PLGA monocomponent scaffolds and bicomponent scaffolds. Owning to the structure similarity between PDLLA and PLGA, peaks at wavenumbers of 2,957, 2,941, 1,759 and 1,088 cm^−1^ were found in all FTIR spectra in Fig. 5 except the spectrum for Ca–P nanoparticles. The peaks appearing at 2,957 and 2,941 cm^−1^ could be attributed to asymmetric stretching vibration and symmetric stretching vibration of C–H. Peaks at 2,356 cm^−1^ could be assigned to the asymmetric stretching (ν_3_ mode) of CO_2_. And the peaks appearing at 1,759, 1,634 and 1,088 cm^−1^ could be attributed to C=O stretching vibration, C=O stretching vibration and C–O stretching vibration, respectively. Compared to the spectra for raw materials, no FTIR peak shift was found for PLGA and PDLLA fibers in electrospun scaffolds.

**Fig. 6:**
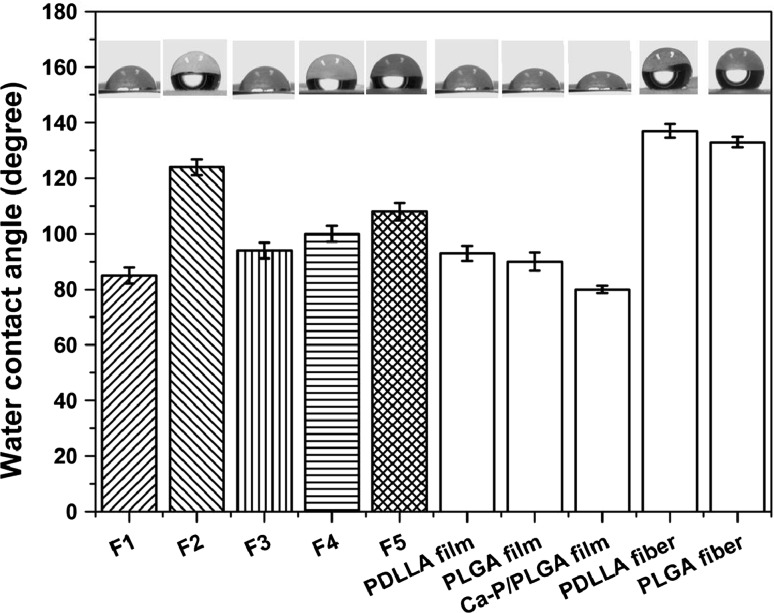
Water contact angle of electrospun scaffolds and solvent-cast films

The wettability of electrospun fibrous scaffolds plays an important role in the biological performances of scaffolds, affecting cell attachment, adhesion and proliferation, and was thus characterized by measuring the water contact angle in this investigation. Figure 6 displays the results obtained: the contact angle measurements, and the contact angle images. Scaffolds electrospun from PDLLA or PLGA polymer solutions exhibited very high water contact angles (135.0 ± 4.5° and 133.0 ± 3.3°, respectively), which were much larger than those of polymeric films produced by the solvent-cast method. This phenomenon was also reported by other researchers [34, 35]. With the incorporation of 10wt% of Ca–P nanoparticles, Ca–P/PLGA nanocomposite scaffolds had a slightly lower water contact angle than PLGA polymer scaffolds. For the two types of monocomponent scaffolds, emulsion electrospun F1 scaffolds were hydrophilic with a water contact angle of 85.4 ± 4.3° whereas nanocomposite F2 scaffolds were hydrophobic with a water contact angle of 127.7 ± 3.6°. The bicomponent scaffolds were all hydrophobic and with an increase in the amount of Ca–P/PLGA nanocomposite fibers, the water contact angle of biocomponent scaffolds increased gradually.

### Encapsulation and in vitro release of rhBMP-2

The encapsulation efficiency (EE) of rhBMP-2 was determined for mono- and bicomponent scaffolds using Eq. (1). Scaffolds F1, F3, F4 and F5 had the rhBMP-2 encapsulation efficiency of 86.4 ± 3.2 %, 88.4 ± 2.8 %, 85.6 ± 3.4 % and 86.7 ± 2.1 %, respectively, and no significant difference was found (*p* > 0.05) among the scaffolds, suggesting that in this investigation, the EE value of rhBMP-2 was not affected by the variation of bicomponent scaffold composition (the component ratio).

**Fig. 7:**
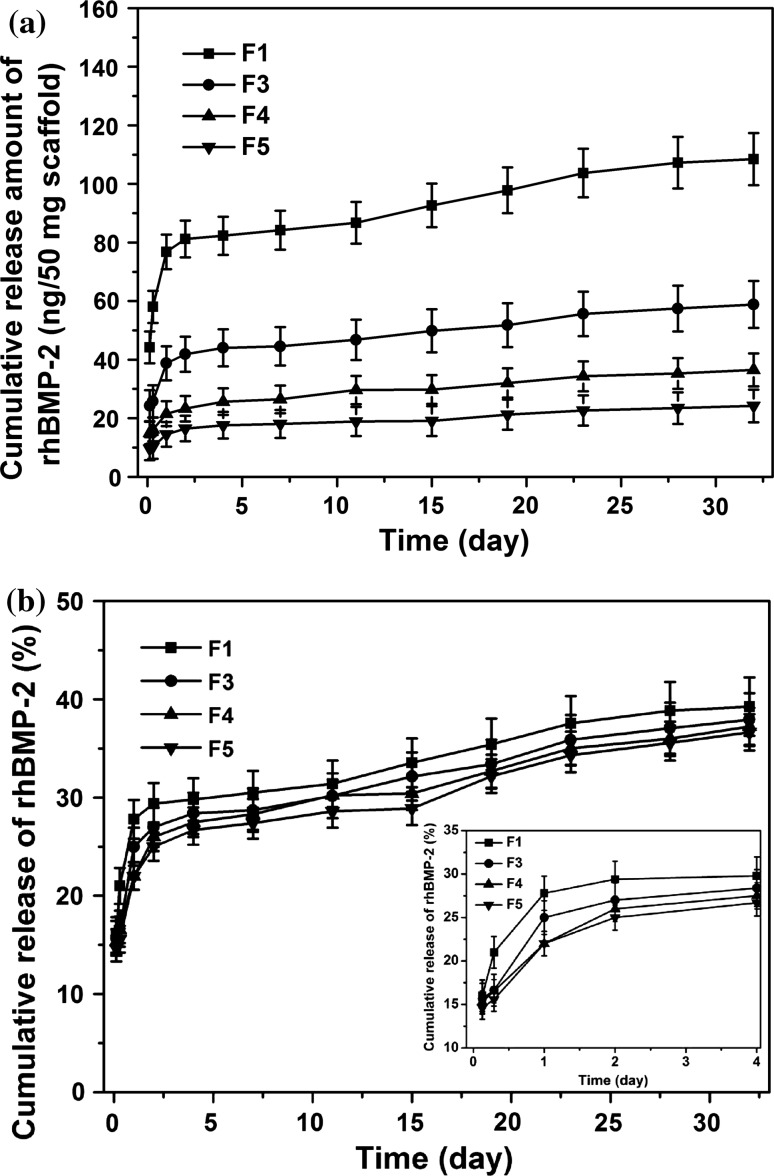
In vitro release behaviour of rhBMP-2 from mono- and bicomponent scaffolds: **a** cumulative release amount with time, **b** cumulative release percentage with time

The in vitro rhBMP-2 release curves for mono- and bicomponent scaffolds are shown in Fig. 7. The in vitro release amounts of rhBMP-2 from bicomponent scaffolds with different component ratios were clearly different over the release test period of 32 days (Fig. 7a). After 32 days of in vitro release, the ratios of the released amount of rhBMP-2 from F3, F4 and F5 scaffolds (at 55.4 ± 3.2, 34.3 ± 2.5 and 22.6 ± 2.7 ng) to the released amount from F1 scaffolds (at 108.5 ± 4.3 ng) was 1:(1.97 ± 0.31), 1:(3.12 ± 0.51) and 1:(4.70 ± 0.50), following the same trend as that of the mass ratio of the rhBMP-2/PDLLA component to the total bicomponent scaffolds [1:(0.97 ± 0.02), 1:(1.74 ± 0.06) and 1:(2.54 ± 0.08), respectively]. These results suggested that using DSDPES and controlling the fibrous component ratio, scaffolds with different rhBMP-2 loading and hence different rhBMP-2 release amount (during release and at the end of release) could be achieved, tailoring the release dosage for individual clinical applications. To further study the release behaviour, the release curves were also plotted in terms of release percentage for the test period (Fig. 7b). In plotting these cumulative in vitro release curves for rhBMP-2, the actual amounts of rhBMP-2 encapsulated in respective types of scaffolds were used as 100 % and the amounts of cumulatively released rhBMP-2 from scaffolds at the time points were divided by the actual encapsulated amounts. It was evident that for F1, F3, F4 and F5 scaffolds, there was an initial burst release within the first 24 h, followed by a much slower and sustained release of rhBMP-2. At 24 h, the initial burst release from F1 scaffolds (the monocomponent rhBMP-2/PDLLA scaffolds) led to a cumulative release level of 27.0 ± 0.7 % of the rhBMP-2 encapsulated, which was slightly higher than the release levels of 20.3 ± 1.5 % to 26.2 ± 1.4 % exhibited by bicomponent scaffolds. After 32 days, the cumulative release of rhBMP-2 reached the 39.2 ± 3.7 % (108.5 ± 4.3 ng) level for F1 scaffolds while the release levels of 37.5 ± 2.3 %, 36.8 ± 2.2 % and 35.7 ± 2.0 % were found for F3, F4 and F5 scaffolds (the bicomponent scaffolds) (*p* > 0.05), respectively, indicating that the release of rhBMP-2 was independent of the component ratio of bicomponent scaffolds.

**Fig. 8:**
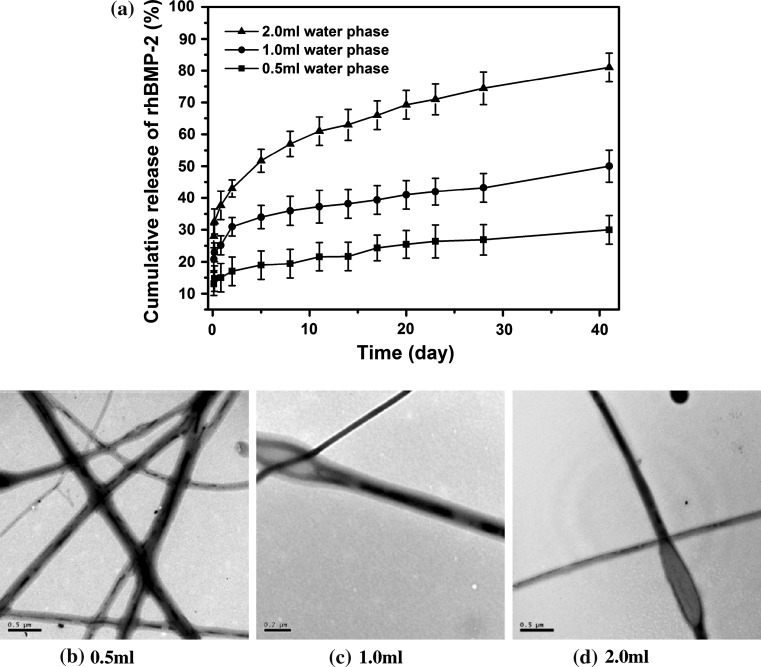
Effects of water phase volume in emulsion on the fiber core–shell structure and rhBMP-2 release from bicomponent scaffolds: **a** in vitro release behaviour of rhBMP-2 from bicomponent scaffolds, and **b**, **c** and **d** TEM micrographs of rhBMP-2/PDLLA fibers produced from emulsions with water phase volumes

The above results suggested that while using DSDPES and controlling the fibrous component ratio could be advantageous for constructing bicomponent scaffolds with different rhBMP-2 loading levels (and also Ca–P loading levels), but the rhBMP-2 release profile was not affected. For further modulating the release profile of rhBMP-2, changes could be made to the rhBMP-2/PDLLA component while keeping the Ca–P/PLGA component unchanged. Therefore, in this investigation, the water phase volume was varied to study the effects of emulsion composition on the release of rhBMP-2 from bicomponent scaffolds, with another two types of scaffolds being made (F6 and F7 scaffolds, Table 1). It can be seen from Fig. 8a that, compared to F3 scaffolds which was electrospun from emulsions with the intermediate water phase volume (1.0 ml), F6 scaffolds made with a lower water phase volume (0.5 ml) exhibited a smaller burst release (reaching the 16.4 ± 3.3 % level after 24 h), which was followed by a sustained release to the 28.5 ± 4.5 % release level after 32 days. On the contrary, when the water phase volume was raised to a higher level (2.0 ml) in emulsions for electrospinning the F7 scaffolds, a larger burst release that reached the 30.2 ± 4.4 % release level at 24 h was observed for F7 scaffolds, which was followed by a sustained release to the 55.7 ± 5.2 % release level after 32 days. TEM examination of rhBMP-2/PDLLA fibers in F3, F6 and F7 scaffolds showed that increasing the water phase volume in emulsions would increase the continuity of the core and also the ratio between the diameter of the core and the whole fiber of emulsion electrospun fibers (Fig. 8b). For instance, when the water phase volume increased from 0.5 to 1.0 ml and 2.0 ml, the core diameter-to-fiber diameter ratio increased significantly from 1:(5.6 ± 0.4) to 1:(3.1 ± 0.3) and further to 1:(2.0 ± 0.2) (*p* < 0.05). The variation of shell thickness in core–shell structured fibers would affect the rhBMP-2 release behaviour. In a parallel research, our experiments demonstrated that increasing the polymer concentration in emulsions would lead to smaller burst release as well as reduced burst release level at 24 h and the total release amount at 32 days.

### In vitro scaffold degradation

**Fig. 9:**
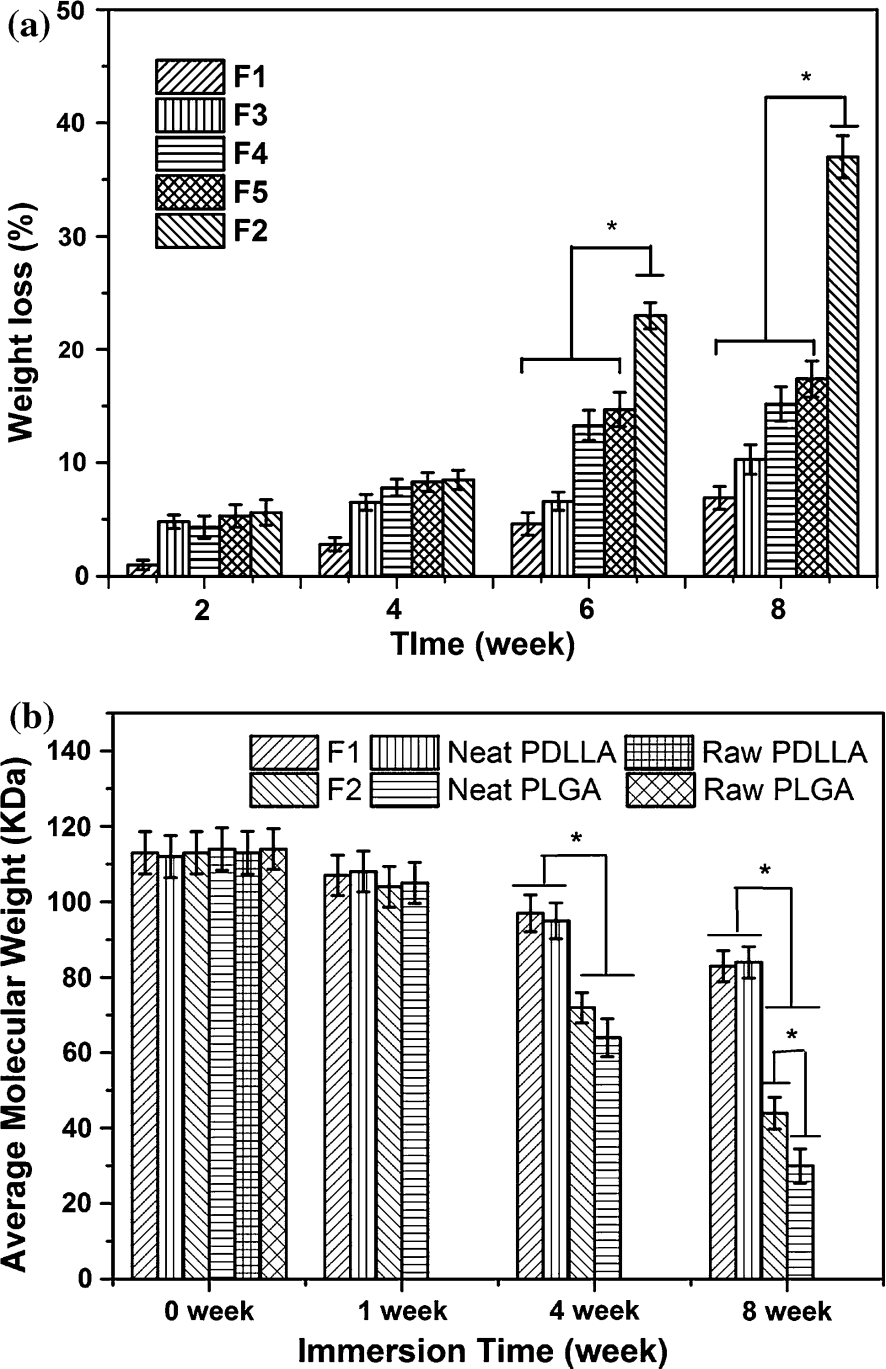
In vitro degradation of mono- and bicomponent scaffolds. **a** Weight loss of scaffolds, and **b** decrease in average molecular weight of fiber polymer matrix

The mass loss of scaffolds (F1 to F5 scaffolds) during the in vitro degradation test period was monitored and the results are summarized in Fig. 9a. All scaffolds exhibited an increased mass loss with increasing incubation time. After 4 weeks in vitro degradation, F1 scaffolds had only 3.1 ± 0.6 % mass loss, less than that of other scaffolds (7.5 ± 1.0, 8.0 ± 1.0, 8.2 ± 0.9 and 8.7 ± 0.9 % for F3, F4, F5 and F2 scaffolds, respectively). After 8 weeks in vitro degradation, the mass loss between F1, F3, F4, F5 and F2 scaffolds was significant (*p* < 0.05). F2 scaffolds exhibited a high mass loss of 35.3 ± 3.3 % after 8 weeks while F1 scaffolds only had a 7.0 ± 1.1 % mass loss. And bicomponent scaffolds (F3 to F5 scaffolds) having a higher proportion of the Ca–P/PLGA fibrous component showed a larger mass loss, but the mass loss was not proportional to the mass ratio between Ca–P/PLGA fibers and rhBMP-2/PDLLA fibers. The results indicated that by adjusting the component ratio, the degradation behavior of bicomponent scaffolds may be controlled.

The decrease in molecular weight was also monitored for monocomponent F1 and F2 scaffolds, neat PDLLA scaffolds, neat PLGA scaffolds and raw polymers (PDLLA and PLGA). It can be seen from Fig. 9b that statistically there was no difference among as-fabricated F1 scaffolds, neat PDLLA scaffolds and raw PDLLA polymer for the polymer average molecular weight (at the time point of 0 week), indicating that the short emulsion preparation process and electrospinning process did not have a negative effect on the average molecular weight of emulsion electrospun scaffold. The average molecular weight was not statistically different among the raw PLGA polymer, neat PLGA scaffolds and as-fabricated F2 scaffolds either. After 4 and 8 weeks in vitro degradation, F1 scaffolds (with PDLLA as the matrix for fibers) exhibited only small reductions in average molecular weight (from 112.0 ± 6.2 kDa to 97.0 ± 6.4 kDa and 88.0 ± 4.4 kDa). When comparing the average molecular weight between F1 scaffolds and neat PDLLA scaffolds (the control) over the degradation test period, no significant difference was found. In comparison, F2 scaffolds showed significant reductions in average molecular weight (from 112.0 ± 6.5 kDa to 72.0 ± 7.2 kDa and 43.0 ± 5.3 kDa) after 4 and 8 weeks in vitro degradation. Furthermore, the decrease in average molecular weight of F2 scaffolds (with PLGA as the matrix for fibers) was less than that of neat PLGA scaffolds.

**Fig. 10:**
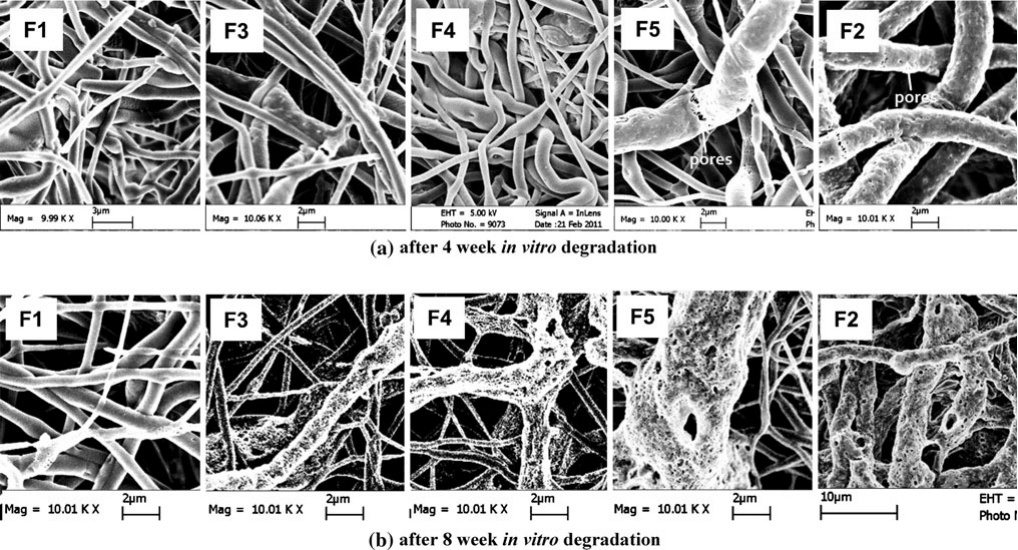
Morphology of fibers and structure of mono- and bicomponent scaffolds after in vitro degradation: **a** after 4 weeks degradation, and **b** after 8 weeks degradation

The morphology and porous structure of mono- and bicomponent scaffolds (F1 to F5 scaffolds) were examined under SEM at different time points during in vitro degradation tests. As shown in Fig. 10, after 4 weeks immersion in PBS at 37 °C, the fiber diameter of all scaffolds (F1 to F5 scaffolds) increased, exhibiting a swelling phenomenon. The porous structure of scaffolds had changed significantly as compared to the original scaffold structure (Fig. 4) and differed among different type of scaffolds. For instance, while F1 scaffolds still showed a relatively smooth fiber surface, fibers in F2 scaffolds had a rough surface with numerous nanopores. The differences among scaffolds after in vitro degradation may be explained by the different degradation rates of polymers for fibers. However, the morphological changes of Ca–P/PLGA fibers in bicomponent scaffolds were not as large as the changes for fibers in monocomponent Ca–P/PLGA scaffolds (the F2 scaffolds). After 8 weeks degradation, no large change in morphology or structure was observed for F1 scaffolds. But F2 scaffolds and the Ca–P/PLGA fibers in bicomponent scaffolds (F3 to F5 scaffolds) showed further and significant degradation with much larger pores and more nanopores on the fiber surface, making the surface much rougher.

## Discussion

### Structure and properties of mono- and bicomponent scaffolds

Due to the similarity of nanofibrous structure to that of ECM of human body tissues, which could induce excellent cell response, and also the capability of providing controlled delivery of drug and/or biomolecules, electrospun biodegradable fibrous scaffolds, especially nonwoven mats, have been widely employed for tissue regeneration applications. The research of using electrospun scaffolds in tissue engineering has now shifted to exploring multifunctional fibrous scaffolds which can, for example, deliver growth factors for enhancing tissue formation. To incorporate different types of species (ceramic nanoparticle, drug, protein, etc.) in fibers, different electrospinning techniques may be utilized in order to avoid/minimize potential problems. Emulsion electrospinning, which can form core–shell structured fibers, can be employed to form fibrous vehicles to deliver delicate, easily denatured biomolecules such as growth factors.

Different from coaxial electrospinning which forms core–shell structured fibers from the very beginning of jet thinning stage of electrospinning, the formation of the core–shell structured fibers under emulsion electrospinning is a more complicated dynamic process. It is commonly understood that when emulsion jets were electrospun into fibers, the water droplets surrounded by the polymer solution in the jets were stretched into long, continuous water filaments and the paralleled water phase coalesced simultaneously, forming a core–shell structure. However, the core–shell evolution process may not be this simple. Our previous research indicated that the state of the core–shell structure in fibers was closely related to the fiber diameter when the water droplets in emulsions were finally converted into the continuous water phase core of solidified fibers. In our investigation, the water droplets in emulsions had an initial diameter of 3–5 μm; however, after the stretching induced by electrospinning, numerous water droplets with much smaller diameter (about 100–200 nm) were formed. This was because when electrospinning started, the spherical water droplets in emulsions underwent simultaneous deformation and became elliptical water droplets or longer but thinner water columns. Once the electricstatic force was above a critical value, elliptical water droplets and/or thin water columns would break up into a string of smaller water droplets. This breakup phenomenon could be explained by Rayleigh/capillary instability, which was often encountered in immiscible fluid systems, and determined by several factors such as viscosity ratio between the water phase and oil phase, radius of the water phase, interfacial tension, etc. Different from closed biphasic fluid systems, fibers made by electrospinning underwent rapid solvent evaporation; therefore, the real-time shape variations of the water phase within fibers could be “frozen” by the solidified shell layer. Due to the slow velocity of solvent ventilating from thick fibers, deformed water droplets or instable water columns had sufficient time to break up into smaller droplets; whereas in very thin fibers, much quicker solvent ventilating resulted in the solidification of the shell layer before the breakup of the water phase, forming a longer and more continuous water phase core. In the meantime, the coalescence of parallel water droplets and water filaments in the transverse direction of fibers during solidification of emulsion jets was also found to be affected by the fiber diameter: a larger extent of water phase coalescence occurred in thinner fibers rather than in thick fibers. The inward movement of the water phase to the center of fibers could be explained by (1) dielectrophoresis, which contributes to the mutual attraction between electriferous water droplets, and (2) the “evaporation and stretching induced de-emulsification” theory, which pointed out that the viscosity difference between the water droplets and polymer solution in emulsion jets would direct the water phase to settle into the interior of fibers instead of on the fiber surface [28]. The above discussions suggest that the fiber diameter had played a key role in determining the state of the core–shell structure in emulsion electrospun fibers, and both the fiber diameter and the state of the core–shell structure would have significant effects on the release profile of biomolecules encapsulated. In this investigation, ultrafine PDLLA emulsion electrospun fibers (with a diameter of 570 ± 130 nm) were obtained, which had a single water phase core and a continuous core–shell structure, and hence were employed as a delivery vehicle for rhBMP-2.

The cell adhesion and growth on a material surface or a scaffold is strongly influenced by the substrate wettability. Many studies have suggested that cells adhere, spread and grow more easily on moderately hydrophilic substrates than on hydrophobic or very hydrophilic substrates [36]. Results from our wettability tests showed that compared to flat, non-porous films made by the solvent-casting method, fibrous scaffolds fabricated by electrospinning had much larger water contact angles. This phenomenon was caused by the rough surface of scaffolds. On fibrous scaffolds, the test water droplet could not easily penetrate into inter-fiber pores in the rough surface of hydrophobic fibrous membranes. Therefore, the water droplet was merely supported on a semi-solid and semi-air plane surface, resulting in a significant increase in the water contact angle. A similar observation was made by Ma et al. [35] who found that the water contact angle of electrospun polyethylene terephthalate (PET) fibrous membranes was significantly larger than that of PET film. Furthermore, it was also reported that scaffolds electrospun from emulsions formulated with the help of a surfactant had much smaller water contact angle [37], and this was similarly observed in our investigation. For the electrospun Ca–P/PLGA scaffolds, the incorporation of Ca–P nanoparticles reduced the water contact angle, thus improving the scaffold wettability. This was similarly observed for composite scaffolds incorporated with other nano-sized bioceramic particles [23]. The affinity of calcium phosphate bioceramics (in either bulk or particulate form) to water contributed to the improved wettability of composite scaffolds containing bioceramic nanoparticles.

In the current study, to form bicomponent scaffolds with different mass ratios of the two components, multiple syringes connected with individual needles were used as the solution supplies for electrospinning the Ca–P/PLGA fibrous component. By applying a high voltage on the needles of parallel syringes, nanocomposite suspension jets were ejected, which subsequently formed the Ca–P/PLGA fibrous component in bicomponent scaffolds (F2 to F5 scaffolds). SEM micrographs in Fig. 3f show that the Ca–P/PLGA fibers were evenly distributed in the bicomponent scaffolds, indicating the success of the DSDPES technique and the multiple-syringe strategy for varying the composition of bicomponent scaffolds. Results listed in Table 2 suggest that using the DSDPES technique and our experimental setup, the composition of bicomponent scaffolds could be controlled. There were slight differences between nominal compositions and measured, real compositions of bicomponent scaffolds, which were mainly due to electrostatic repulsion induced fiber loss. (The electrostatic repulsion problem, which is a general issue in the electrospinning field, is tackled currently by our investigations into a new electrospinning technique.) In future studies, through the minor adjustment of number of syringes, intended compositions for bicomponent scaffolds could be achieved. An empirical relationship could be established between the number of syringes (as solution supplies) and composition of bicomponent scaffolds [30, 31].

### In vitro release behavior of rhBMP-2 from mono- and bicomponent scaffolds

Because months are needed for bone tissue regeneration in the body, employing PDLLA as the polymer matrix for rhBMP-2/PDLLA fibers could achieve a prolonged release of rhBMP-2. In this investigation, a sustained release of rhBMP-2 following an initial burst release was found for scaffolds containing rhBMP-2/PDLLA fibers. As discussed in the previous section on the formation of core–shell structured fibers during emulsion electrospinning, the inward movement of water droplets played a crucial role in forming the core–shell structure. Although the majority of water droplets could move inward to achieve their coalescence in the interior of fibers, the rest of them which were on or close to the surface of the emulsion jet initially may be stayed away from the fiber core due to rapid solvent evaporation, forming pockets of encapsulated rhBMP-2 near fiber surface. Therefore, rhBMP-2 localized on these sites would initially diffused out of fibers because these close-to-surface pockets had very thin shells which favored rhBMP-2 diffusion, resulting in its burst release. Owing to the slow degradation of PDLLA in scaffold, after the initial burst release, all scaffolds exhibited a slow but sustained release of rhBMP-2 at a constant rate up to 32 days, which could be attributed to the slow diffusion of rhBMP-2 from the inner water phase core, through the polymer matrix in fibers or the aqueous pores therein, to the immersion medium [25]. Since the composition and electrospinning conditions of the rhBMP-2/PDLLA component in bicomponent scaffolds were maintained the same, no statistic difference in the in vitro rhBMP-2 release was found among mono- and bicomponent scaffolds, suggesting that the variation of component ratio did not affect the rhBMP-2 release profile. The fiber morphology and scaffold weight remained relatively unchanged in the first month of in vitro degradation, indicating that diffusion is the dominant mechanism for rhBMP-2 release, instead of fiber degradation.

To analyze the diffusional release of rhBMP-2, an emulsion electrospun scaffold could be modeled as a polydispersion of cylinders. The transport mechanism for rhBMP-2 could be treated as the case of a monodispersion of cylinders. The equation describing the transport of drugs from nonswellable one-dimensional cylindrical devices is [25]:

4 $$ \frac{{M_{t}}}{{M_{\infty}} } = kt^{n} $$

where *M*_*t*_ is the mass of drug released at the time *t*, *M*_*∞*_ is the mass of drug released as time approaches infinity, *k* is a constant and *n* is the diffusion exponent. Since electrospun fibers have very high aspect ratios (in the current investigation, the circumference of the rotating drum for fiber collection was 31.4 cm and the average fiber diameter was around 570 ± 130 nm and it could be assumed that the fiber was not broken for at least one rotation of the drum), the release of rhBMP-2 from nanofibers fabricated by emulsion electrospinning could be assumed to be one-dimensional diffusion [25]. With calculations, the electrospun rhBMP-2/PDLLA scaffold gave

5 $$ \frac{{M_{t}}}{{M_{\infty}} } = 6.52\,t^{0.35} $$

with a correlation factor of *R* ^2^ = 0.945. According to Ritger and Peppas [38], for one-dimensional Fickian diffusion of drugs from a monodispersion of cylinders,

6 $$ \frac{{M_{t}}}{{M_{\infty}} } = kt^{0.45} $$

The deviation of the diffusional exponent obtained in this investigation (*n* = 0.35) from 0.45 may be explained as: (1) the rhBMP-2/PDLLA fibers had a distribution of fiber diameters, not a single, uniform fiber diameter; (2) the dissolution of the protein may have constituted an additional barrier; and (3) some (only a small percentage) of the fibers may have been flattened, assuming non-cylindrical shapes.

The current investigation has demonstrated that rhBMP-2 could be released in a sustained manner from emulsion electrospun rhBMP-2/PDLLA fibers in mono- or bicomponent scaffolds. However, improvements could be made in future investigations by using different strategies for the current rhBMP-2 release profiles consisting of an initial burst release (to the 20–27 % release levels) and a subsequent sustained release (to the 36–39 % release levels after 32 days). Varying the emulsion composition (for example, changing the water phase volume in the current investigation) can be an effective way to modulate the release profile for rhBMP-2. With increasing water phase volume, larger initial burst release and higher release levels after sustained release could occur. Electrospinning of emulsions with larger water phase volumes could form more isolated water phase pockets near the fiber surface, resulting in higher burst release. Meanwhile, fibers electrospun from emulsions with larger water phase volumes had thinner shells and smaller fiber diameters, favoring the diffusion of rhBMP-2 from the water phase core of fibers, resulting in higher release levels during sustained release. On the contrary, electrospinning of emulsions with smaller water phase volumes produced fibers with larger diameters and thicker shells, leading to not only smaller burst release but also lower release levels at 42 days. Using another biodegradable polymer of a different degradation rate or a polymer blend as the fiber matrix is another strategy for modulating the release of rhBMP-2 from emulsion electrospun fibers. Our current investigation suggested that, sustained release of biomolecules at a constant rate over a certain portion of the release time could be achieved. Well-defined core–shell structures could be produced for emulsion electrospun fibers for the sustained release. Future investigations will be conducted into achieving better controlled release and release levels of rhBMP-2 from the scaffolds.

The bioactivity of growth factors released from electrospun scaffold is an important issue. Ekaputra et al. [39] studied the bioactivity of vascular endothelial growth factor (VEGF) after 48 h release from electrospun fibers. Compared with the same amount of VEGF which had not undergone the fiber fabrication process, VEGF released from electrospun scaffolds after 48 h retained above 80 % bioactivity. Schofer et al. studied the influence of poly(l-lactic acid) (PLLA) nanofibers and BMP-2-containing PLLA nanofibers on the growth and osteogenic differentiation of human MSCs. They found that there was no initial down-regulation of the gene expression of alkaline phosphatase (ALP), osteocalcin, and Collagen-I if BMP-2 was directly incorporated into PLLA for obtaining nanofibers via electrospinning, indicating that growth factors such as BMP-2 could undergo the electrospinning process and maintain its bioactivity [40]. Therefore, in the current investigation, the bioactivity of encapsulated rhBMP-2 could be well preserved.

### In vitro degradation of mono- and bicomponent scaffolds

In vitro degradation of polymer-based fibrous scaffolds depends on many factors, including the polymer itself, fiber structure, fiber diameter, bioceramic phase in composite fibers, scaffold porosity, scaffold size, pH of the medium, etc. It has been well accepted that acidic hydrolysis is the main mechanism for the degradation of polyesters such as PDLLA and PLGA. The autocatalytic degradation process comprises a fast degradation onset and a delayed, yet even more powerful polymer erosion when the polymer becomes water soluble, which is due to the reduction of the molecular weight during degradation. In this investigation, apart from creating multifunctional scaffolds, DSDPES in combination with the use of multiple-syringe solution supplies proved to provide a useful strategy to modulate the degradation behavior of scaffolds by employing two types of polymers with different degradation rate as fiber matrices (PLGA degradation: 1–2 months, and PDLLA degradation: 12–14 months). As shown in Fig. 9a, all scaffolds exhibited less than 5 % weight loss within the first 2 weeks of in vitro degradation. Although PLGA was hydrated already and began to degrade (PLGA with a LA:GA ratio of 50:50 began to degrade after 10 days of immersion in PBS [41]), the scaffolds still maintained their integrity. The weight loss of scaffolds containing Ca–P/PLGA fibers could be attributed to the initial dissolution of Ca–P from the nanocomposite fiber surface and the initial PLGA polymer degradation. The much higher weight loss of F2 scaffolds (the monocomponent Ca–P/PLGA scaffolds) after 6 weeks in vitro degradation could be attributed to the erosion of PLGA fiber matrix. At this stage of in vitro degradation, large amounts of LA and GA monomers came out of the PLGA polymer matrix, making the immersion liquid (PBS) much more acidic and consequently accelerated the dissolution of Ca–P nanoparticles in composite fibers. In contrast, F1, F3, F4 and F5 scaffolds showed much less weight loss than F2 scaffolds within the same degradation period as they did not contain (in the case of F1 scaffolds) or contained much less Ca–P/PLGA fibers. The much smaller decrease in average molecular weight of F1 fiber compared to that of F2 fiber polymer matrix (Fig. 9b) suggested that the hydrolysis of rhBMP-2/PDLLA fibers was slower than that of Ca–P/PLGA fibers within the first 4 weeks of in vitro degradation. Meanwhile, since F3 to F5 bicomponent scaffolds had less amounts (in mass) of Ca–P/PLGA fibers than F2 monocomponent scaffolds, less amounts of LA and GA monomers were released to the immersion medium of F3 to F5 scaffolds during in vitro degradation, which rendered the medium less acidic than that of medium for F2 scaffolds, leading to less weight loss due to PLGA hydrolysis and Ca–P dissolution. Compared to electrospun neat PLGA scaffolds, the incorporation of Ca–P nanoparticles into PLGA-based scaffolds had already caused a reduced decrease in molecular weight loss. Even though acidic degradation products such as LA and GA would decrease the pH value of immersion liquid, the released alkaline ions from the Ca–P nanoparticles in composite fibers during in vitro degradation could compensate the decrease in pH and consequently slow down the creation of an otherwise more acidic environment, leading to a reduced molecular weight loss. This phenomenon was also observed by other researchers [21, 42].

The morphological changes of mono- and bicomponent scaffolds during in vitro degradation were closely related to the degradation process. As shown in Fig. 10, after 4 weeks of degradation and in comparison with Ca–P/PLGA fibers in bicomponent scaffolds, much more enlarged pores appeared on the surface of fibers of F2 scaffolds. The formation of micro- or nanopores on these fibers could be attributed to the dissolution of incorporated Ca–P nanoparticles in the fibers and also the erosion of fiber polymer matrix. It was suggested by other researchers that the release rate of calcium and phosphate ions from composite scaffolds was controlled by the acidic products generated by the polymer matrix degradation [43]. Therefore, the immersion medium of F2 scaffolds was the most acidic after 4 weeks of scaffold degradation, causing quicker dissolution of Ca–P particles and simultaneously an intensive auto-catalytic hydrolysis and therefore the formation of more and larger pores on fiber surface. After 8 weeks of in vitro degradation, Ca–P/PLGA fiber in all scaffolds exhibited similar degraded morphology due to the intensive effects of matrix polymer erosion and Ca–P dissolution and some of the fibers collapsed into fragments of various sizes, whereas rhBMP-2/PDLLA fibers in the scaffolds still maintained an intact fibrous structure. Compared to electrospun neat PLGA fibers, although the weight loss of Ca–P/PLGA fibers was larger, the incorporation of Ca–P nanoparticles to form fibrous nanocomposite scaffolds by electrospinning could reduce, to some certain extent, the molecular weight decrease of matrix PLGA polymer in scaffolds. This observation was in agreement with research results obtained by other researchers from their composite scaffolds [41]. These results from our investigations as well as from others’ research imply that in addition to the strong influence of the component ratio of bicomponent scaffolds, the degradation behaviour of bicomponent scaffolds could be further tailored by the addition of Ca–P nanoparticles and by controlling the Ca–P amount in scaffolds.

## Conclusions

The osteoinductive growth factor rhBMP-2 and osteoconductive Ca–P nanoparticles could be incorporated in electrospun fibrous PDLLA scaffolds and PLGA scaffolds via emulsion electrospinning and conventional electrospinning, respectively. The continuous core–shell structure was formed in emulsion electrospun rhBMP-2/PDLLA nanofibers and most of rhBMP-2 was encapsulated in the water phase core of fibers. For Ca–P/PLGA nanocomposite fibers, using our processing method, Ca–P nanoparticles were well dispersed in electrospun fibers. Through DSDPES and with the use of multiple syringes to supply solutions (or emulsions) for electrospinning, novel bicomponent scaffolds with controlled fibrous component ratios could be constructed and each fibrous component could be evenly distributed in the scaffolds. When the component ratio was varied for bicomponent scaffolds comprising rhBMP-2/PDLLA fibers and Ca–P/PLGA fibers, the amount of rhBMP-2 released from bicomponent scaffolds could be varied but all bicomponent scaffolds exhibited a similar rhBMP-2 release profile. The release of rhBMP-2 from scaffolds could be further modulated by altering the emulsion composition such as water phase volume for emulsion electrospinning. The in vitro degradation study revealed that bicomponent scaffolds with different component ratios had different degradation behaviours. This investigation has successfully demonstrated the construction of multifunctional bicomponent scaffolds through DSDPES and the capability to control scaffold composition (the fibrous component ratio). The novel bicomponent scaffolds could provide balanced osteoinductivity and osteoconductivity, sustained rhBMP-2 release and controlled scaffold degradation for bone tissue engineering.
